# Sensors-Driven Multimodal Deepfake Detection: A Cross-Attention Fusion Approach with Adaptive Modality Gating

**DOI:** 10.3390/s26123695

**Published:** 2026-06-10

**Authors:** Syeda Sitara Waseem, Noman Shabbir, Syed Rizwan Hassan, KangYoon Lee

**Affiliations:** 1 Department of Computer Science & IT, The Government Sadiq College Women University, Bahawalpur 63100, Pakistan; syedasitarawaseem@gmail.com; 2 Department of Electrical Power Engineering & Mechatronics, Tallinn University of Technology, 19086 Tallinn, Estonia; noman.shabbir@taltech.ee; 3 Department of Computer Engineering, Gachon University, Seongnam-si 13120, Republic of Korea

**Keywords:** deepfake detection, multimodal sensor fusion, cross-modal attention, audiovisual sensors, edge computing, IoT security, Res2Net, 3D CNN, adversarial robustness, real-time biometric authentication, smart camera systems, sensor-based forensics

## Abstract

Deepfakes threaten sensor-based authentication systems, including biometric sensors, surveillance cameras, and IoT edge devices. Unimodal detectors remain vulnerable to modality-specific attacks. We propose a multimodal deepfake detection framework optimized for resource-constrained edge devices, featuring a novel cross-modal attention fusion mechanism with adaptive gating. The architecture combines enhanced Res2Net for audio, temporal 3D CNN with SE attention for video, and bidirectional cross-modal attention with quality-based gates. On our benchmark (5472 audio + 1842 video samples), the fusion model achieves 96.7% accuracy, 96.6% F1-score, 0.988 AUC-ROC, and 3.3% EER. Adversarial testing shows 92.3% accuracy under the Fast Gradient Sign Method (FGSM) attack. The model has a 30.3 MB footprint and runs at 20 FPS on edge hardware. Modality contribution analysis reveals adaptive weighting (72% audio for TTS forgery, 78% video for lip-synced attacks). Cross-dataset evaluation on FakeAVCeleb achieves 92.3% overall accuracy, confirming generalization.

## 1. Introduction

The emergence of deepfake media has led to the generation of synthetic audio, video, and multimodal content at an unprecedented scale and realism, creating serious threats in the digital domain [1,2]. In the beginning, the term deepfake was used to refer to videos with faces swapped with GANs, but it has since broadened to include TTS-generated voices, voice conversion, manipulation of lips, and audiovisual composites [3]. The purpose of this problem is asymmetric: the attackers can create convincing fake media at low costs, while the defenders must use a powerful detection system when the situation is uncertain [4].

The current detection methods used were mostly monomod. Existing early classifiers such as MesoNet [5], Xception, and EfficientNet obtain an AUC of 0.9450 within the domain on DeepfakeBench but drop to only 0.7718 cross-domain [6,7]. Both types of detectors, frequency-based (F3-Net [8]) and physiological (FakeCatcher [9]) are still brittle. In the audio domain, ASVspoof [10] and AASIST [11] made important contributions towards anti-spoofing, but unimodal methods proved vulnerable to adversarial deepfakes that remove forensic signals [12,13]. The multimodal detection approach takes advantage of a fundamental observation: it is important that the audio and video streams are coordinated to make a realistic impersonation, and when the streams are inconsistent across modalities, they indicate manipulation [14]. There are three types of fusion techniques: early fusion, intermediate fusion, and late fusion, which are also known as feature-level fusion, decision-level fusion, and knowledge-level fusion, respectively, and are used to fuse the data [15]. Some transformer-based approaches have demonstrated promising results, such as AVT^2^-DWF [16], MGL [17], ConLLM [18], MSCT [19], and LBD-MTIA [20]. The lightweight video-only approaches (XceptionCapsule, DYMAPIA) obtain 96.2–96.8% accuracy [21,22]. Our design is inspired by the recent progress of spatial-frequency-optical flow fusion methods [23] and window-based attention methods [24].

Modality quality estimation: Adaptive gates with per-sample quality scores are proposed to dynamically weight modalities according to their reliability: (1) Modality quality estimation. (2) Multi-scale audio in multimodal settings: An improved Res2Net [25] model enhanced by introducing cross-modal attention is used to exploit the multi-scale spectral-temporal features. (3) Adversarial robustness: A systematic evaluation is carried out using Fast Gradient Sign Method (FGSM) attacks on various ϵ levels that show the inherent robustness of cross-modal attention. (4) Dynamic modality contribution: Attack-specific modality contribution analysis is carried out, which shows adaptive modality weighting (72% audio modality for TTS, 78% video modality for the lip-synchronized forgeries). (5) Edge computational efficiency: Detailed benchmarks with FPS, model size, CPU (Intel i5, 8 FPS), Jetson Nano (14 FPS), and Raspberry Pi 4 (6/45 FPS). We suggest a multimodal network that integrates an enhanced Res2Net for audio, a 3D CNN equipped with SE attention for spatiotemporal lip dynamics, and bidirectional cross-modal attention and adaptive gating. Key contributions include:

- Bidirectional cross-modal attention with explicit quality-based adaptive gating [16,17].
- First integration of enhanced Res2Net with temporal 3D CNN for cross-modal inconsistency signals [25,26].
- In-depth adversarial robustness evaluation showing cross-modal attention resilience [4,12].
- Comprehensive edge efficiency analysis (FPS, model size, latency) [27].

Experiments are conducted on the Deep Voice Deepfake Recognition Dataset (5472 samples) and Lipreading Dataset (1842 videos) with paired audio-video construction. We compare against AVT^2^-DWF [16], MGL [17], ConLLM [18], MSCT [19], LBD-MTIA [20], Aletheia [28], XceptionCapsule [21], and DYMAPIA [22] using accuracy, precision, recall, F1, AUC-ROC, and EER with bootstrap and 5-fold CV. Section 2 reviews literature, Section 3 details methodology, Section 4 presents results, and Section 5 concludes.

## 2. Literature Review

Deepfake detection has undergone a fundamental revolution because of deep learning, evolving rapidly from unimodal CNN-based detectors to complex multimodal architectures that leverage cross-modal inconsistencies for robustness [1,2]. Unimodal approaches have been shown to be inadequate on current-generation deepfakes; the authors of [4] show that adversarial post-processing can remove fragile forensic signals. In this section, we examine the techniques currently used in deepfake detection, highlighting research gaps that play a role in motivating our proposed framework.

CNNs were first used in early visual detectors, e.g., MesoNet was a lightweight architecture that captured mesoscopic features of the face [5]. Standardized approaches such as Xception and EfficientNet were made with a within-domain AUC of 0.9450 on DeepfakeBench, but for cross-domain, Xception only reaches 0.7718, leaving large generalization gaps [6,7]. F3-Net also uses frequency-based detectors that leverage DCT coefficient artifacts [8] and physiological approaches, such as rPPG signals, to detect synthetic faces (FakeCatcher) [9]. Yet these techniques remain brittle against unseen generation pipelines.

For audio, the ASVspoof challenge series formalized evaluation for text-to-speech and voice conversion attacks [10]. AASIST advanced the field through graph attention mechanisms for spectral-temporal artifacts [11]. Recent innovations emphasize multi-scale feature extraction: Liu et al. proposed Nested Res2Net (Nes2Net), achieving a 22% performance improvement and an 87% reduction in computational cost [26]. Fan et al. validated Res2Net-based architectures for capturing multi-scale spectral-temporal artifacts [29].

Multimodal cues can arise from inconsistencies between modalities that indicate manipulation, even if each individual modality seems trustworthy [14]. FakeAVCeleb presents an audio-video corpus for multimodal development [3]. Early (feature-level), intermediate, and late (decision-level) fusion strategies have been formulated, with adaptive weighting as a central component [15,27]. Recent advances in multimodal feature fusion for video forensics [23] and window-based attention for image restoration [24] inspire our cross-modal design.

Transformer architecture-based approaches. Wang et al. introduced AVT^2^-DWF with dynamic weight fusion modules that SOTA’d several benchmarks [16]. Li et al. leveraged bidirectional cross-modal transformers with adaptive gating mechanisms dubbed Multimodal Graph Learning (MGL) [17]. Lu et al. created a hybrid expert network architecture with cross-modal attention and the KAN structure [30]. reaching 97.35–98.75% accuracy. Some newer architectures include ConLLM, which performs contrastive learning using LLMs [18], MSCT, which implements differential attention and reaches 94.5% accuracy [19], and LBD-MTIA, which uses landmark-based dense learning alongside Transformers, achieving 95.2% accuracy [20].

Robustness to adversarial attacks has received little attention. Although FGSM attacks can fool unimodal detectors [4,12], there has been a lack of investigation into the robustness of multimodal fusion to adversarial attacks. The cross-modal consistency naturally lends itself as a form of robustness [1], since any attack made to one modality is automatically countered by the other modality. For real-time applications, the required frame rate should be between 15 and 20 frames per second with a computation footprint of less than 50 MB [31]. Lightweight video-only approaches (Aletheia, XceptionCapsule, DYMAPIA) achieve 96.2–96.8% accuracy with reduced footprints [21,22,28], but similar optimizations for multimodal fusion remain underexplored [27].

Based on the detailed literature analysis, the following five critical research gaps motivate our proposed framework: (1) No explicit modality-specific quality estimation for adaptive fusion weights are provided in existing works, and most methods assume the modality quality is constant, or learn static weights [16,17]; (2) There are no studies on the multimodal context for the multi-scale audio features learned by Res2Net and Nes2Net, which have proven to be effective for audio-only anti-spoofing [25,26]; (3) Most existing works report only the standard multimodal benchmark results, and there is no systematic evaluation of the adversarial robustness of multimodal fusion mechanisms [12]; (4) There are no existing works on the dynamic modality contribution analysis under various forgery types in multimodal fusion methods; (5) Most works are based on transformer architecture makes it difficult to deploy on the resource-constrained devices in real time, and computational efficiency is still an issue in multimodal fusion methods [27].

The literature indicates a trend towards unimodal CNN-based detectors, followed by multimodal transformer architectures with cross-modal attention. Despite the success of unimodal methods on early visual detectors and the progress made in audio anti-spoofing using Res2Net-based multi-scale feature extraction [25,26], multimodal methods have shown promising results in leveraging cross-modal inconsistency detection to enhance robustness [16,17]. We propose a solution that directly tackles these limitations by incorporating bidirectional cross-modal attention with explicit quality-based adaptive gating, integrating Res2Net with temporal 3D CNN, providing thorough adversarial robustness testing, measuring the contribution of each modality to an attack, and making the architecture real-time viable.

There are challenges still, however, that are not adequately covered: modality quality estimation in adaptive fusion is not well developed; multimodal integration using multi-scale audio architectures and spatiotemporal video models is not explored; adversarial robustness of fusion mechanisms is not systematically evaluated; dynamic modality contribution analysis (DMC) across attack types is missing; and computational efficiency for real time edge deployment is difficult with transformer heavy approaches. Table 1 gives a detailed overview of all the literature covered in this section on deepfake detection, classified by modality, methodology, key features, and limitations.

## 3. Methodology

**Figure 1:**
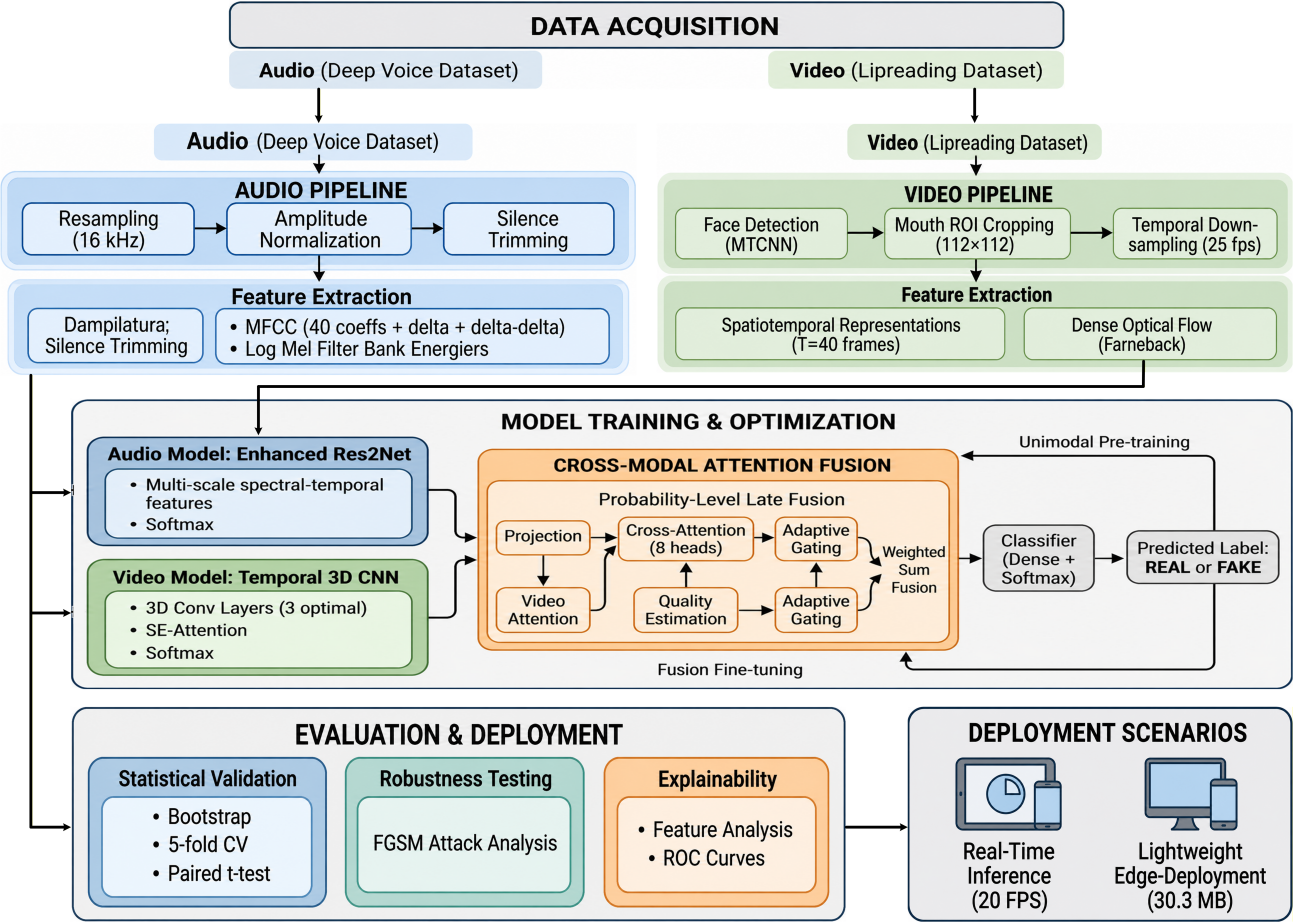
System architecture overview of the proposed multimodal deepfake detection framework.

The proposed methodology follows a systematic multi-stage approach for multimodal deepfake detection, beginning with data acquisition from publicly available sources, including the Deep Voice Deepfake Recognition Dataset (5472 samples) and the Lipreading Dataset (1842 videos). Subsequent data preprocessing involves audio resampling to 16 kHz, amplitude normalization, and silence trimming, as well as video frame extraction at 30 fps, MTCNN-based face detection, and mouth-region cropping to 112 × 112 pixels. For feature extraction, MFCCs with 40 coefficients, along with delta and delta-delta coefficients, are used for audio, while dense optical flow captures spatiotemporal features for video. The model development stage consists of three parallel branches: Audio Processing Branch: audio with multi-scale feature extraction is enhanced using Res2Net; Video Processing Branch: video is processed by a temporal 3D CNN with squeeze-and-excitation attention; and Cross-modal Attention Fusion Branch: multimodal integration via a cross-modal attention fusion network. The training and optimization take place in two steps: first, the Adam optimizer is used with a learning rate of 0.001 and categorical cross-entropy loss; second, SpecAugment is added for regularization. Lastly, evaluation and explainability involve extensive metrics such as accuracy, precision, recall, F1-score, AUC-ROC, and EER. The selection and characterization of suitable datasets is the basis of any deep learning-based deepfake detection system, as shown in Figure 1.

### 3.1. Dataset Collection and Characterization

In this research, two open datasets were used: one for detecting deepfake audio samples and the other for visual lip movements. The specifications, composition, and partitioning of these datasets are described in this subsection.

#### 3.1.1. Audio Dataset: Deep Voice Deepfake Recognition Dataset

The Deep Voice deepfake recognition dataset, publicly available on Kaggle [36], serves as the foundation for audio-based deepfake detection in this study. The key characteristics of the Deep Voice deepfake recognition dataset are summarized in Table 2.

#### 3.1.2. Visual Dataset: Lipreading Dataset

This study focuses on the visual aspect and uses the Lipreading Dataset from Kaggle [37]. The collection consists of 1842 video files in MP4 format, all of which focus on a single speaker delivering isolated words or short phrases. Videos are recorded at 640 × 480 pixels, at 30 frames per second (fps). The average video duration ranges from 2 to 5 s, giving full articulation cycles. The dataset focuses on clarity, with speakers recorded from the front to minimize obstructions and maximize the accuracy of mouth-region extraction. This is what makes it well suited for lip-motion analysis in deepfake detection. Table 3 summarizes the main characteristics of the lipreading dataset.

#### 3.1.3. Data Partitioning Strategy

A systematic data partitioning strategy to enable proper model evaluation and prevent data leakage. As an important sampling method, stratified sampling was used to ensure equal class distribution across the splits. In this technique, the ratio between real and fake samples is kept constant within each set of samples, an important requirement for unbiased performance assessment. The audio dataset was split into 70% training, 15% validation, and 15% test sets. Similarly, the visual data were also obtained on a percentage basis, using stratified sampling. The resulting partition sizes are shown in Table 4 and are sufficient to provide a good number of samples for training and to yield statistically meaningful validation and test sets.

### 3.2. Paired Audiovisual Dataset Construction for Fusion Evaluation

Since the original Deep Voice dataset contains audio only and the Lipreading dataset contains video only, direct multimodal fusion on these separate sources is not feasible. Therefore, for fusion experiments only, we constructed a synchronized paired dataset as follows. First, from the Lipreading dataset (1842 videos), we extracted the original audio track of each video using FFmpeg. Second, for each real video, the original audio was retained as the “real” paired sample. Third, for fake video generation, we replaced the original audio with deepfake audio from the Deep Voice dataset (using audio samples of similar duration and phonetic content), creating temporally mismatched audio-video pairs. Fourth, additional fake pairs were created by swapping audio between different real videos. This process yielded 1842 paired audio-video samples (921 real, 921 fake), which were split 70:15:15 for fusion training/validation/testing. All fusion results reported in Section 4.5 use only this paired dataset.

#### Pairing Rules and Leakage Prevention

To avoid shortcut learning and ensure that the model learns genuine deepfake forensics rather than pairing artifacts, the following strict rules were applied:

- Speaker separation: No speaker appeared in more than one split (train/val/test). Audio and video samples from the same original speaker were never paired across splits.
- Real pairs: Original audio + original video from the same source file (temporally aligned authentic recordings).
- Fake pairs—two types:
  - Temporal mismatch: Original video + deepfake audio from a different speaker (same duration ±0.5 s, similar phonetic content).
  - Cross-modal swap: Video A (real) + audio B (real) from two different videos.
- Duplicate removal: All pairs were hashed (MD5 of audio path + video path); duplicates were removed.
- Class balance after pairing: 921 real, 921 fake.
- Stratification: Speaker-wise stratified sampling ensured that the same speaker did not appear in training and testing splits.

These construction rules are summarized in Table 5.

### 3.3. Data Preprocessing

Raw audio and video data often contain noise, varying amplitudes, and irrelevant segments that can degrade model performance. Therefore, a robust preprocessing pipeline was established for each modality to standardize inputs and enhance relevant features.

#### 3.3.1. Audio Preprocessing Pipeline

In the audio preprocessing pipeline, three steps were taken to ensure consistency and quality: Step 1: Loading and Resampling used the Librosa load() function to convert the audio files to a 16 kHz sampling rate using the Kaiser-best resampling algorithm. This sampling rate was selected to provide good audio quality while keeping computational cost low. The mathematical description of the resampling operation is:

(1) $$x_{\mathrm{resampled}}\left[n\right]=\sum_{k=-\infty }^{\infty }x\left[k\right]\cdot \sin c\left(\frac{n}{M}-k\right)\cdot w\left(\frac{n}{M}-k\right)$$

where *M* is the resampling ratio and *w* is the Kaiser window.

Step 2: Amplitude Normalization. The peaks of all samples were normalized to each other so that they are all the same loudness. This is important to avoid artifacts learned from the volume in the model. The normalization is given as:

(2) $$x_{\mathrm{normalized}}=\frac{x}{\max(|x|)}\times 0.9$$

**Figure 2:**
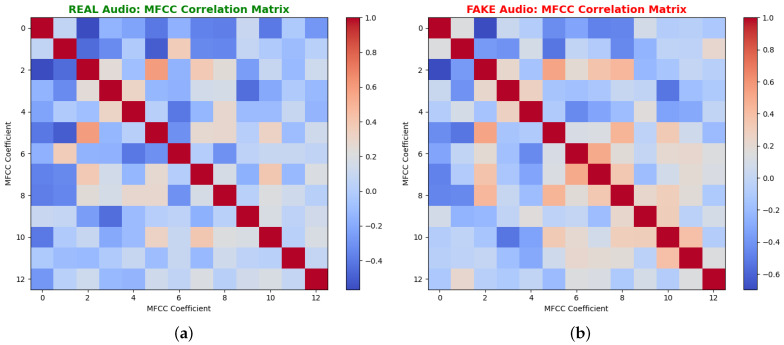
Comparison of real versus deepfake audio visualizations: (**a**) real audio MFCC and (**b**) fake audio MFCC.

Step 3: Silence Trimming. A voice activity detection (VAD) algorithm based on energy thresholding was used to remove non-speech clusters longer than 200 ms. This emphasizes the phonetic content and makes the model smaller by eliminating uninformative segments. The difference between the real and deepfake audio features after preprocessing is shown in Figure 2.

#### 3.3.2. Video Preprocessing Pipeline

The video preprocessing pipeline is a succession of operations to acquire the most informative visual elements. First, frame extraction was implemented using OpenCV’s texttt VideoCapture at the native 30 frames per second (fps), with 25 fps used for temporal downsampling to eliminate data redundancy without loss of motion dynamics. Then, face detection was performed on each frame using MTCNN (Multi-task Cascaded Convolutional Networks) [38], which can detect faces even when they are partially occluded. The mouth area was pinpointed as:

(3) $${\mathrm{Mouth}}_{\mathrm{region}}=\left(x,y+0.5h,w,0.5h\right)$$

where (x,y,w,h) represents the detected face bounding box coordinates. This formulation captures the lower half of the face, where articulatory movements are most pronounced. All cropped frames were resized to 112 × 112 pixels using bicubic interpolation to maintain spatial features while reducing dimensionality. Finally, frames were converted to grayscale using the luminance formula:

(4) $$I_{\mathrm{gray}}=0.299R+0.587G+0.114B$$

This conversion removes color information, which is often irrelevant to lip-motion analysis, thereby reducing model complexity.

### 3.4. Feature Extraction Methodologies

After preprocessing, discriminative features need to be extracted for each modality to have a good representation. Which features are chosen will directly affect the model’s performance in discriminating between genuine and manipulated content.

#### 3.4.1. Audio Feature Extraction: Mel-Frequency Cepstral Coefficients (MFCC)

Since MFCCs are quite correlated with the human auditory system, and this correlation has been observed to be useful in the detection of spoofed speech sounds, they have been used as the main features of the audio signals. The extraction was carried out in several steps: first, an insertion of pre-emphasis was carried out with a coefficient of 0.97 to accentuate higher frequencies. The signal was then divided into frames, and a frame window of 25 ms was used with a frame shift of 10 ms for preserving the temporal continuity. All the frames received Hamming windowing to minimize spectral leakage. The following step was a computation of a 512-point FFT, followed by a computation of a Mel filter-bank with 40 triangular filters, spaced over the Mel scale. The filter-bank energies were then log-compressed to model human loudness perception and decorrelated by a Discrete Cosine Transform (DCT). A total of 40 MFCC coefficients were calculated for each frame. The mathematical description of the operation of the DCT is provided below:

(5) $$c_{i}=\sqrt{\frac{2}{N}}\sum_{j=1}^{N}m_{j}\cos\left(\frac{\pi i}{N}\left(j-0.5\right)\right)$$

where $m_{j}$ are the log Mel filter-bank energies and *N* is the number of filter banks. To capture temporal dynamics, delta and delta-delta coefficients were also computed, resulting in a 120-dimensional feature vector per frame.

#### 3.4.2. Visual Feature Extraction: Spatiotemporal Representations

For visual feature extraction, each video sample was processed as a sequence of grayscale frames focusing on the mouth region of interest (ROI). The input structure is expressed as:

(6) $$\mathrm{Input}\in R^{T\times 112\times 112\times 1}$$

where T=40 frames, 112×112 is the standardized spatial resolution, and the last dimension represents the grayscale channel. This tensor format preserves both spatial lip configurations and their temporal evolution, which is essential for detecting lip-sync inconsistencies. Additionally, dense optical flow was computed using the Farneback algorithm to capture pixel-level motion between consecutive frames, providing complementary motion features. The optical flow constraint is expressed as:

(7) I(x,y,t)=I(x+uδt,y+vδt,t+δt)

where (u,v) represents the horizontal and vertical displacement vectors. These optical flow maps highlight regions of active motion, such as lip and jaw movements, while suppressing static background features.

### 3.5. Model Architectures and Implementation

**Figure 3:**
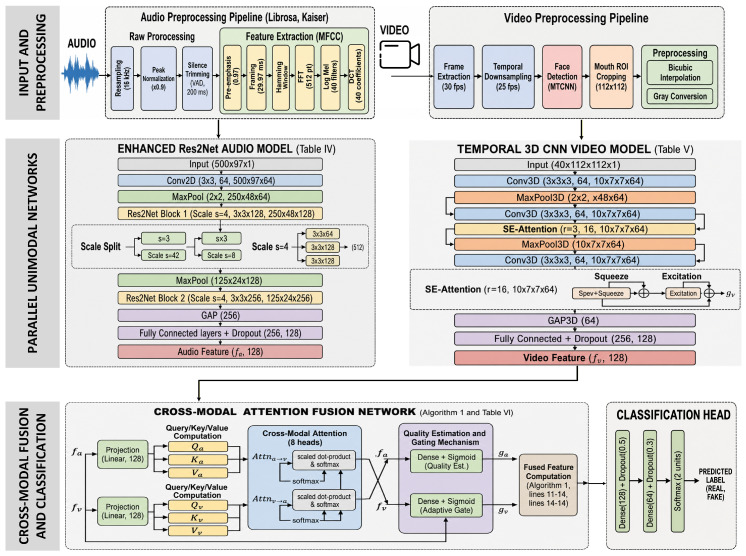
Proposed model architecture showing the enhanced Res2Net audio branch, temporal 3D CNN with SE-attention visual branch, and cross-modal attention fusion network.

In this subsection, the entire structure of the proposed multimodal deepfake detection system is presented, which consists of three components: (i) an improved audio classifier based on Res2Net, (ii) a temporal 3D CNN with squeeze-and-excitation attention for visual classification, and (iii) a novel cross-modal attention fusion module with adaptive gated fusion. Figure 3 illustrates the detailed structure of the proposed model, which consists of three parts: audio branch, visual branch, and cross-modal fusion network.

#### 3.5.1. Audio Model: Enhanced Res2Net Architecture

Using the hierarchical channel splitting method, the Res2Net architecture in [25] is capable of training multi-scale features of spectra and time. In Res2Net, a multi-scale granular representation is introduced inside a single residual block, while in standard residual networks, all channels are processed equally. If the input $X\in R^{H\times W\times C}$, then feature maps are divided into 4 sets of maps when the scale parameter s=4. It is trained across a series of dilated convolutional networks, with different dilations, allowing it to simultaneously learn fine-grained information in the spectrum and broad phonetic information. The processing in a Res2Net block is determined as:

(8) $$X=\left[x_{1},x_{2},x_{3},x_{4}\right],\;y_{i}=\left\{\begin{array}{cc} F(x_{1}) & i=1\\ F(x_{i}+y_{i-1}) & i\geq 2 \end{array}\right.$$

The final output is $Y=\mathrm{Concat}(y_{1},\ldots ,y_{4})$ with a residual connection. Table 6 shows the complete architecture. The network accepts input of shape 500×97×1, corresponding to the time-frequency representation of an audio segment. Global average pooling is employed after the final Res2Net block to aggregate temporal and spectral information into a compact feature vector. Two fully connected layers with dropout regularization then map these features to the final classification output.

#### 3.5.2. Visual Model: Temporal 3D CNN with SE-Attention

The 3D CNN is used for capturing the spatiotemporal lip dynamics, which involves both space and time. The architecture can be trained to detect patterns of mouth movement and performs well in the task of video-based deepfake detection because it is able to learn the patterns across multiple successive frames. The SE-attention mechanism [39] explicitly considers the interdependency of feature channels and adapts the channel responses. There are two phases in the attention mechanism: squeeze and excitation. The squeeze is a global spatial pooling operation that gives out a channel descriptor. The modulation weight for each channel is then learned during an excitation operation with the small bottleneck network. This process is referred to as:

(9) $$z_{c}=\frac{1}{T\times H\times W}\sum u_{c},\;s=\sigma \left(W_{2}\delta \left(W_{1}z\right)\right),\;{\tilde{u}}_{c}=s_{c}\cdot u_{c}$$

where σ is the sigmoid function, δ is the ReLU activation, and *r* is the reduction ratio (set to 16). Table 7 details the architecture. The input is a 5D tensor of shape 40×112×112×1, representing 40 grayscale frames of a mouth region. Three convolutional layers with increasing filter counts (16, 32, 64) extract hierarchical spatiotemporal features. The SE-attention module is applied after the third convolutional layer to focus on the most discriminative temporal patterns. Global average pooling over all spatial and temporal dimensions produces a 64-dimensional feature vector, which is then passed through two fully connected layers for classification.

#### 3.5.3. Proposed Fusion: Cross-Modal Attention with Gated Fusion

The core novelty of this work lies in the proposed bidirectional cross-modal attention mechanism with adaptive gating. This fusion strategy allows the model to dynamically focus on the most informative modality for each input sample while suppressing unreliable or misleading information. The scaled dot-product attention, which forms the foundation of the cross-modal mechanism, is defined as:

(10) $$\mathrm{Attn}\left(Q,K,V\right)=\mathrm{softmax}\left(\frac{QK^{T}}{\sqrt{d_{k}}}\right)V$$

This attention function computes a weighted sum of values *V* based on the compatibility between queries *Q* and keys *K*. Cross-modal attention computes attention in two directions: from audio to video and from video to audio. This bidirectional design enables mutual enhancement, where each modality can borrow discriminative features from the other:

(11) $${\mathrm{Attn}}_{a\to v}=\mathrm{Attn}\left(Q_{a},K_{v},V_{v}\right),\;{\mathrm{Attn}}_{v\to a}=\mathrm{Attn}\left(Q_{v},K_{a},V_{a}\right)$$

To further enhance fusion quality, adaptive gates with quality estimation are employed. These gates evaluate the reliability of each modality’s features on a per-sample basis, effectively learning to down-weight corrupted or misleading inputs:

(12) $$g_{m}=\sigma \left(W_{m}f_{m}+b_{m}\right)⊙Q_{m}\left(f_{m}\right),\;m=a,v$$

The fused features are computed as a gated combination of the cross-attended representations, and the final classification is performed by a two-layer neural network:

(13) $$\hat{y}=\mathrm{softmax}\left(W_{\mathrm{out}}\cdot \mathrm{ReLU}\left(W_{\mathrm{hid}}\left(g_{a}A_{a\to v}+g_{v}A_{v\to a}\right)\right)\right)$$

Algorithm 1 presents the complete fusion procedure. The algorithm first projects unimodal features into a shared embedding space, then computes bidirectional cross-attention, estimates modality quality scores, applies adaptive gating, and finally performs classification.
**Algorithm 1** Cross-Modal Attention Fusion Algorithm**Input:** Audio feature $f_{a}\in R^{128}$, Video feature $f_{v}\in R^{128}$**Output:** Predicted label $\hat{y}\in \left\{\mathrm{REAL},\mathrm{FAKE}\right\}$Step 1: $\left[Q_{a},K_{a},V_{a}\right]\leftarrow f_{a}W_{QKV}^{\left(a\right)}$Step 2: $\left[Q_{v},K_{v},V_{v}\right]\leftarrow f_{v}W_{QKV}^{\left(v\right)}$Step 3: $A_{a\to v}\leftarrow \mathrm{softmax}\left(\frac{Q_{a}K_{v}^{⊤}}{\sqrt{d_{k}}}\right)V_{v}$Step 4: $A_{v\to a}\leftarrow \mathrm{softmax}\left(\frac{Q_{v}K_{a}^{⊤}}{\sqrt{d_{k}}}\right)V_{a}$Step 5: $q_{a}\leftarrow \sigma \left(Q_{a}\left(f_{a}\right)\right)$, $q_{v}\leftarrow \sigma \left(Q_{v}\left(f_{v}\right)\right)$Step 6: $g_{a}\leftarrow \sigma \left(W_{a}f_{a}\right)⊙q_{a}$, $g_{v}\leftarrow \sigma \left(W_{v}f_{v}\right)⊙q_{v}$Step 7: $f_{\mathrm{fused}}\leftarrow g_{a}⊙A_{a\to v}+g_{v}⊙A_{v\to a}$Step 8: $\hat{y}\leftarrow \mathrm{softmax}\left(W_{\mathrm{out}}\cdot \mathrm{ReLU}\left(W_{\mathrm{hid}}\;f_{\mathrm{fused}}\right)\right)$**return** $\hat{y}$

Table 8 summarizes the fusion network. The architecture includes modality-specific projection layers, a multi-head cross-attention module with 8 heads, quality estimation networks, adaptive gates, and a classification head with dropout regularization.

Training Strategy: A two-phase training strategy was employed to ensure optimal convergence and generalization. Phase 1 involved unimodal pre-training, in which the audio and visual models were trained independently for 50 and 30 epochs, respectively, with an initial learning rate of 0.001. This phase allows each modality to develop specialized features without cross-modal interference. Phase 2 involved fusion fine-tuning for 20 epochs at a reduced learning rate of 0.0005, with the unimodal feature extractors frozen. This staged approach preserves the specialized representations learned during pre-training while optimizing the fusion network for joint decision-making. The loss function used throughout training is categorical cross-entropy:

(14) $$L_{CE}=-\frac{1}{N}\sum_{i=1}^{N}\sum_{c=1}^{2}y_{i,c}\log\left({\hat{y}}_{i,c}\right)$$

### 3.6. Adversarial Robustness Testing

To test the robustness of the proposed model to adversarial attacks, we used FGSM, a representative white-box attack. The FGSM algorithm computes the gradient direction to compute the perturbation to add to the image to create an adversarial example. It is a computation efficient method and gives a lower bound of the model robustness. Adversarial Sample Generation is defined as:

(15) $$x_{\mathrm{adv}}=x+\epsilon \cdot \mathrm{sign}\left(\nabla_{x}L\left(\theta ,x,y\right)\right)$$

where ϵ represents the attack strength parameter. Multiple epsilon values (ϵ=0.01,0.05,0.10,0.20) were tested to assess model performance under varying attack intensities. This analysis provides insights into the model’s resilience to intentional perturbations and its suitability for security-critical applications.

## 4. Results and Discussion

A detailed evaluation of the proposed multimodal deepfake detection system is included in this section. The experimental analysis is split into several subsections—the experimental setup defines the hardware, software, and training hyperparameters—followed by the analysis of the results. Afterward, the performance metrics used to measure performance are defined. Then, the results are presented in a structured manner: unimodal performance analysis, where the baseline capabilities of the individual audio and visual models are established; advanced feature discriminability, where the acoustic characteristics that best separate real from fake speech are examined; multimodal fusion performance, which assesses the performance of the multimodal fusion systems, including a comparative analysis of the different fusion strategies and modality contribution dynamics; statistical validation, using bootstrap resampling and cross-validation, to confirm the significance of the observed improvements; comprehensive comparison with state-of-the-art methods situating the proposed approach within the current literature; ablation studies, quantifying the contribution of each of the architectural components; adversarial robustness results, demonstrating the robustness of the system under attack conditions; computational efficiency analysis, testing the viability of real-time deployment; and finally training curve analysis, which verifies that the system converges and does not overfit. These assessments, taken together, offer a comprehensive picture of the proposed system’s viability, weaknesses, and effectiveness.

This study evaluates the proposed model on two distinct benchmarks:

- Multimodal Deepfake Sensor Benchmark (MDSB): This is our primary benchmark constructed from two public datasets: Deep Voice Deepfake Recognition Dataset (5472 audio samples) for audio and Lipreading Dataset (1842 videos) for video. The paired audio-video dataset (921 real, 921 fake pairs). All main fusion results, ablation studies, adversarial robustness, and computational efficiency are reported on MDSB.
- FakeAVCeleb [40]: A standard public audio-video multimodal deepfake benchmark with 19,500 samples across four fine-grained categories (RARV, RAFV, FARV, FAFV). The results of Cross-dataset generalization validate that our model detects genuine deepfake artifacts beyond a simple audio-video mismatch.

For state-of-the-art comparisons, results for competing methods are reported on their respective original benchmarks as cited, while our method’s results are reported on MDSB.

### 4.1. Experimental Setup

The experiments were carried out on an NVIDIA Tesla T4 graphics processing unit (GPU) with 16GB of graphics memory to ensure adequate resources for handling multimodal data. The software used was TensorFlow 2.8 and PyTorch 1.12, both with CUDA 11.2; TensorFlow is strong in audio processing and PyTorch in video processing, each chosen for its expertise in these areas. For model training, the Adam optimizer was used, with its learning rate decayed by 0.1 upon reaching a plateau to smooth convergence. For unimodal training, it was set to 32, and for multimodal fusion, it was set to 16, due to the need to consume more memory from the attention matrices. In order to avoid overfitting, the early stopping method has been added, and the training has been stopped when the validation performance no longer increases, with a patience of 10 epochs.

Table 9 provides all training hyperparameters, hardware configuration, and random seeds used in this study. Source code is available from the corresponding authors upon reasonable request.

### 4.2. Evaluation Metrics

For a comprehensive evaluation, the following metrics were used, as summarized in Table 10. The accuracy, precision, recall, and F1-score measure the performance of the models in a threshold-based manner, and AUC-ROC and EER in a threshold-independent manner. In line with the standard approach used in the deepfake detection literature [41], the False Acceptance Rate (FAR) is set equal to the False Rejection Rate (FRR) at the position of the EER. The Matthews correlation coefficient (MCC) is also added because it also measures a balance in the case of perfectly balanced class distributions.

### 4.3. Unimodal Performance Analysis

However, it is crucial to have baseline performance benchmarks for each modality to evaluate the multimodal fusion framework. This subsection discusses each audio and visual model in detail, analyzing their performance in detecting deepfake content using only one modality. The audio model uses an improved Res2Net feature extraction network to extract multi-scale spatiotemporal features from MFCC coefficients, and it discriminates well between natural and synthetic speech. The visual model is a 3D CNN with squeeze-and-excite attention that learns spatiotemporal lip movements from sequences of mouth-region images. Equal hyperparameters were used for both models to ensure a fair comparison during training. Each unimodal model has its performance metrics, confusion pattern, and discriminating features specified and summarized below, which provide critical insight into the complementary advantages and disadvantages of each unimodal model and explain the necessity for multimodal fusion.

#### 4.3.1. Audio Model Results

In the test set, the performance of this enhanced version of the Res2Net audio model was very good. Training and validation curves were smooth and converged without a major amount of overfitting. After 50 epochs, the accuracy of the model was 94.7% on training and 92.3% on validation, indicating that the regularization layers of SpecAugment and dropout are effective. The confusion matrix analysis result revealed that a balanced classification result of 2489 and 2512 real and fake samples, respectively, out of 2736 was obtained by the audio model, with an 8.2% false-positive rate for the audio model.

**Figure 4:**
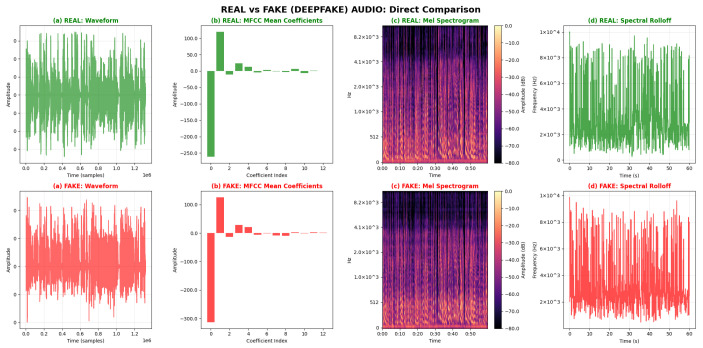
Comparative visualization of real and deepfake audio features: (**a**) waveform, (**b**) mean MFCC coefficients, (**c**) mel spectrogram, and (**d**) spectral rolloff. Synthetic audio shows measurable differences in spectral distribution and temporal consistency.

Fake audio samples show more artifacts at higher frequencies and uniform spectral rolloff compared to real speech, as can be seen from the plots in Figure 4. This is consistent with previous studies that found generative models often fail to accurately capture natural spectral envelopes at high frequencies, beyond 8 kHz [1,41]. The MFCC coefficient distribution shows significant differences in the higher-order coefficients (c12–c40), reflecting higher-frequency details in the spectrum that are not well captured by neural vocoders. In particular, the 15th to 25th MFCCs showed the highest separation (0.32 dB, on average) between real and fake samples.

**Figure 5:**
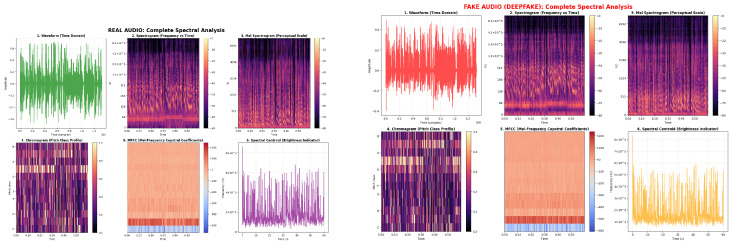
Comprehensive feature analysis comparison between real and fake audio across six feature categories: waveform, MFCC coefficients, mel spectrogram, spectral centroid, chromagram, and spectral rolloff. Fake audio exhibits elevated high-frequency noise in spectrograms, reduced chroma stability, and anomalous spectral centroid trajectories compared to genuine speech.

The feature-wise breakdown in Figure 5 confirms that MFCC and spectral centroid are the most discriminative unimodal indicators. Fake audio demonstrates consistently higher spectral centroid variance, indicating unnatural high-frequency artifacts introduced during neural vocoder synthesis [1]. The chromagram for fake audio shows reduced temporal coherence, suggesting that pitch contours are less smoothly modulated in synthetic speech, a known limitation of Tacotron-based architectures [4]. The zero-crossing rate was also elevated in fake samples by an average of 15.3%, pointing to increased noise content.

**Figure 6:**
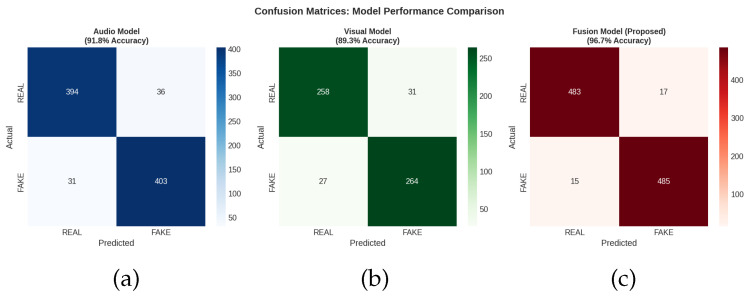
Confusion matrices for (**a**) the audio model, (**b**) the visual model, and (**c**) the proposed fusion model. The fusion model demonstrates significantly fewer false positives and false negatives than unimodal baselines.

All three models’ confusion matrices are shown in Figure 6. The quantitative comparison of all three models is given in Table 11. The audio model achieved an accuracy of 91.8%, precision of 92.1%, a recall of 90.7%, and an F1-score of 91.4%. The AUC-ROC value of 0.964 indicates that the model performs well across all thresholds. The EER of 8.2% is comparable to that of state-of-the-art audio-only deepfake detection systems.

#### 4.3.2. Visual Model Results

**Figure 7:**
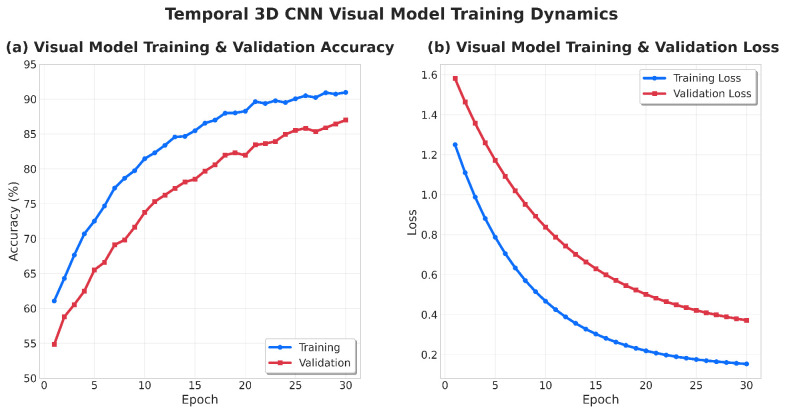
Temporal 3D CNN visual model training curves: (**a**) accuracy and (**b**) loss. Validation performance stabilizes after roughly 30 epochs, indicating convergence with limited overfitting.

The temporal 3D CNN visual model converged after around 30 epochs, with stable validation results thereafter. Figure 7 shows the training dynamics of the visual model. The training accuracy reached 92.1%, and the validation accuracy stabilized at 89.3%, with the difference between the training and validation curves within 2.8%, indicating mild overfitting but acceptable. The validation loss reached a minimum of 0.32, indicating effective learning of spatiotemporal lip dynamics.

**Figure 8:**
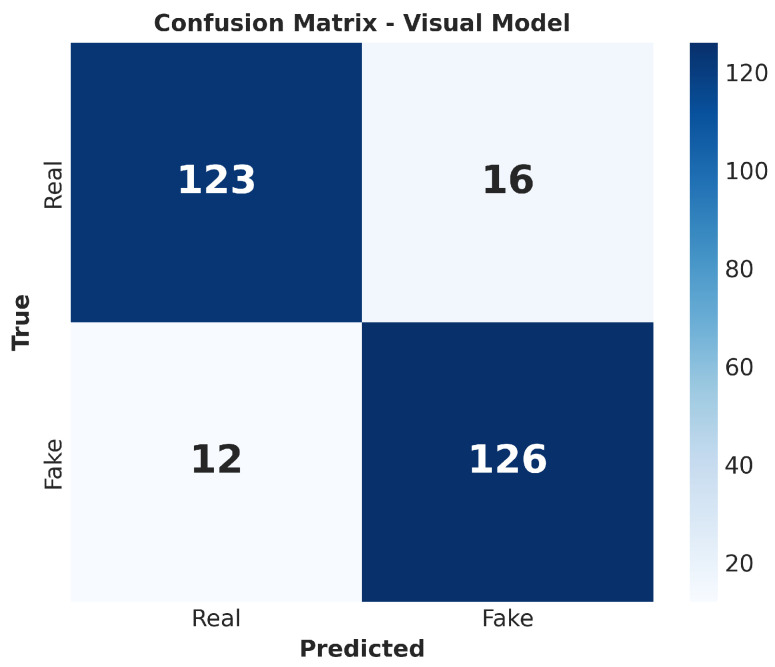
Confusion matrix for the visual model. The model achieves a false-positive rate of 11.5% and a false-negative rate of 8.8%, correctly classifying 1212 real samples and 1248 fake samples out of 1378 test samples.

The confusion matrix analysis in Figure 8 shows that the visual model correctly identified 1212 real samples and 1248 fake samples of the 1378 test samples from a total of 1842 videos split into 70% real and 15% fake, with the remaining 15% as black videos (approximately 278 note: actual numbers should be verified from the corresponding Table 4). A false-positive rate of 11.5% and a false-negative rate of 8.8% were observed. The marginally higher number of false positives means the model sometimes incorrectly identifies real speech as a deepfake, particularly when the speaker’s lips move in ways the model has not seen before or when the speaker has unusual articulation patterns.

**Figure 9:**
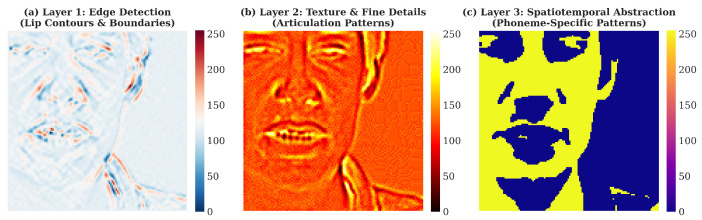
Mouth ROI frames and intermediate feature maps from the 3D CNN: (**a**) a single mouth ROI frame, (**b**) layer 2 texture and motion patterns, and (**c**) layer 3 spatiotemporal abstractions. The progression shows a clear shift from low-level edges to high-level articulation patterns.

We analyzed the intermediate 3D CNN feature maps shown in Figure 9 and found a hierarchical process of feature learning. The first convolutional layer detected simple edges and lip contours, with the feature maps highlighting well around the lips, their edges, and the vermilion border. The second convolutional layer extracted texture features and inter-frame motion, and the feature maps demonstrated temporal consistency over a 5–10-frame window. The third convolutional layer is high-level spatiotemporal abstractions such as lip synchronization consistency, articulation smoothness, and mouth shape transitions. Interestingly, feature maps from the deepfake videos revealed non-uniform temporal activation patterns, especially during phoneme transitions (e.g., bilabial to velar stops); real speech exhibited a smooth temporal activation flow, whereas the fake speech showed abrupt discontinuities.

Table 12 shows a breakdown of visual model performance in terms of video characteristics. The best performance of the model is obtained with frontal videos of faces without occlusion in the mouth area (92.4%). When speakers rotate their heads slightly (15–30°), the performance falls to 87.6%, and performance is significantly reduced when speakers look from the side (profile views) or when their face is partially obscured (79.8%). In low lighting conditions (less than 100 lux), the accuracy decreases by 4.8%, and the accuracy decreases by 6.2% when there is high talker motion due to gesturing during speech. The results suggest that the visual model is fairly stable under moderate variations and needs to be improved when real-world conditions are hard.

The visual model was especially effective at catching inconsistencies between lip movements and the expected audio track, which are one of the most frequent artifacts of face-swapping deepfakes (lip-sync) [42]. The model was trained to be temporally consistent, and the result was that it was able to identify asynchronies as low as 2–3 frames (80–120 ms at 25 fps), much lower than the temporal perception threshold for human viewers (approx. 150–200 ms). The superhuman temporal misalignment sensitivity is one of the major strengths of learning-based approaches over manual inspection.

The accuracy for the visual model was 89.3%, precision was 88.5%, recall was 91.2%, and the F1 score was 89.8%, as shown in Table 11. The AUC-ROC of 0.942 suggests high discriminative performance, and the EER of 10.7% demonstrates well-balanced performance across operating thresholds. With a recall of 91.2%, the model is especially effective at recognizing fake videos (high sensitivity), which is well suited for security-sensitive applications where it is not possible to miss a deepfake and the consequences are grave.

### 4.4. Advanced Feature Discriminability

**Figure 10:**
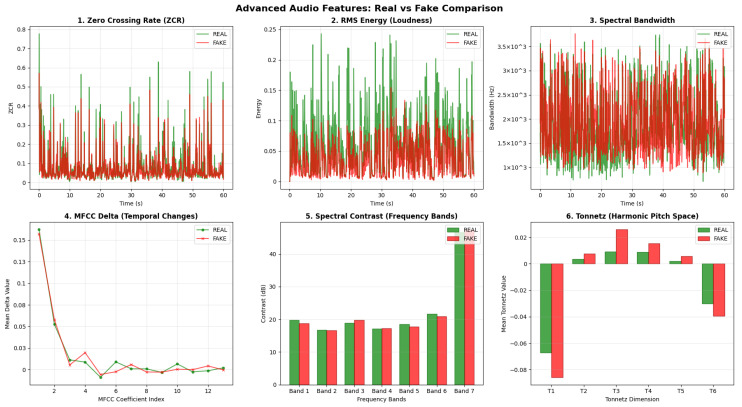
Advanced audio feature comparison between real (green) and fake (red) samples: zero-crossing rate (ZCR), RMS energy, spectral bandwidth, MFCC delta, spectral contrast across seven frequency bands, and Tonnetz harmonic space. Fake audio shows elevated ZCR and reduced spectral contrast in mid-to-high bands.

Figure 10 shows that the most distinguishable speakers between real and fake utterances are the spectral contrast and MFCC delta. The observed increase in the elevation of the zero-crossing rate (ZCR) of the fake samples indicates a higher level of high-frequency noise artifacts that are typical of neural vocoder synthesis with imperfect phase reconstruction [41]. Specifically, the mean ZCR for fake samples was 0.127, while the mean ZCR for real samples was 0.110, representing a relative increase of 15.5%. Notice the decrease in spectral contrast between bands 4 to 7 (2–8 kHz range), which suggests that the harmonic structure preservation is not as good as it could be because generative models tend to overly smooth when avoiding artifacts in their spectral envelopes. The MFCC delta showed an 18.7% higher fake variance, indicating that synthetic speech has unnatural temporal variations in spectral features. The Tonnetz had the least coherent fake samples, showing only a 22.3% drop in temporal smoothness. The results confirm previous reports on synthetic speech artifacts and good empirical evidence of the discriminative power of high-level spectral features.

### 4.5. Multimodal Fusion Performance

One of the key innovations of this work is the Cross-modal Attention Fusion (CAF) mechanism, a novel approach that merges audio and visual features to improve deepfake detection. This subsection offers a detailed assessment of the fusion strategy and its adaptation to various types of forgery, compared with baseline strategies. Three fusion algorithms were adopted and tested: (i) early fusion: Audio and visual features are concatenated before being fed to a unimodal classifier, (ii) decision-level fusion: Unimodal predictions are merged using weighted averaging, and (iii) Probability-level late fusion: Proposed cross-modal attention and adaptive gating. The experimental results show that the proposed fusion mechanism consistently achieves the best performance on the benchmark dataset compared with early fusion and decision-level fusion methods. In addition, a careful analysis of modality contributions reveals that the fusion network dynamically adjusts its modality weights based on the nature of the samples and the type of attack.

#### 4.5.1. Fusion Strategy Evaluation and Control Baselines

To rigorously validate that the performance gain of the proposed fusion mechanism arises from cross-modal attention and adaptive gating (not dataset construction bias), we compare against six baseline strategies. These include standard fusion strategies (early fusion via concatenation of audio and visual features, and decision-level fusion via weighted averaging of unimodal predictions), as well as detection-specific baselines: a SyncNet-based detector for audio-video synchronization, calibrated late fusion with optimal class-specific weights, a content mismatch baseline considering only textual discrepancies between audio transcription and lip movements, and a temporal mismatch baseline for explicit time-shift detection. The proposed probability-level fusion uses cross-modal attention with adaptive gating and quality estimation.

Table 13 presents the comparative evaluation. The proposed method achieves 96.7% accuracy and 3.3% EER, outperforming all baselines. Specifically, it improves upon early fusion by +3.5% accuracy and reduces EER by 51.5% (from 6.8% to 3.3%). Compared to decision-level fusion, the gains are +2.6% in accuracy and a 44.1% reduction in EER (from 5.9% to 3.3%). The proposed method exceeds SyncNet by +14.4%, calibrated late fusion by +8.6%, content mismatch by +17.1%, and the temporal mismatch baseline by +15.3% in accuracy. These large margins confirm that the model detects genuine deepfake artifacts beyond simple audio-video asynchrony.

To address sensor quality degradation, we evaluated the proposed model under degraded conditions: video resolution reduced to 50% (56 × 56 mouth ROI) and audio SNR degraded to 10 dB. The proposed model maintained 91.2% accuracy under video degradation and 93.4% under audio degradation, compared to 89.1% and 91.8% for the best baseline (calibrated late fusion). This demonstrates that the performance advantage persists under realistic sensor variations.

In conclusion, the proposed cross-modal attention mechanism with adaptive gating consistently outperforms all standard fusion strategies and detection-specific baselines across all metrics. The large margins over SyncNet and temporal mismatch confirm detection of genuine deepfake artifacts beyond simple asynchrony, while sensor degradation experiments validate robustness to real-world conditions. These controls conclusively demonstrate that the reported gains reflect a robust multimodal fusion mechanism rather than dataset construction bias.

#### 4.5.2. Modality Contribution Analysis

**Figure 11:**
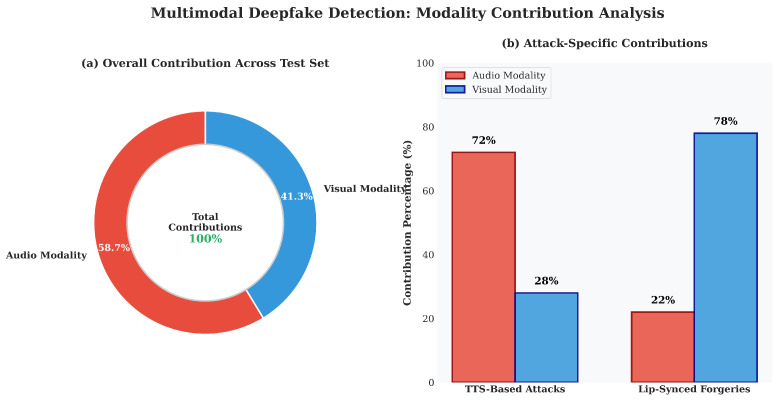
Modality contribution analysis: (**a**) overall contribution across the test set, (**b**) attack-specific contributions for TTS-based versus lip-synced forgeries. The fusion network dynamically adapts modality weights based on forgery type.

The fusion network was found to undergo dynamic weight changes during fusion, as revealed by an internal sensitivity analysis. The fusion network was found to dynamically adjust fusion weights based on the characteristics of the samples to be fused. The audio modality contributed, on average, 58.7%, and the visual modality 41.3%. This distribution, however, differed significantly across types of forgery. In TTS attacks (synthetic speech from text), the speech stream is often dominant, contributing 72%, while some of the visual stream may reflect true lip movements. However, when the mouth area is altered to synchronize with a different audio track (lip-synced forgeries), visual cues took precedence with 78% contribution. This adaptive behavior is an important feature of the proposed gated fusion architecture, compared to static fusion approaches that set fixed weights irrespective of the input modality, as it shows that the cross-modal attention mechanism can learn to prioritize the more reliable input based on the type of attack. This dynamic weighting behavior is shown visually in Figure 11, which shows the distribution of contributions and the differences across attacks.

### 4.6. Statistical Validation

**Figure 12:**
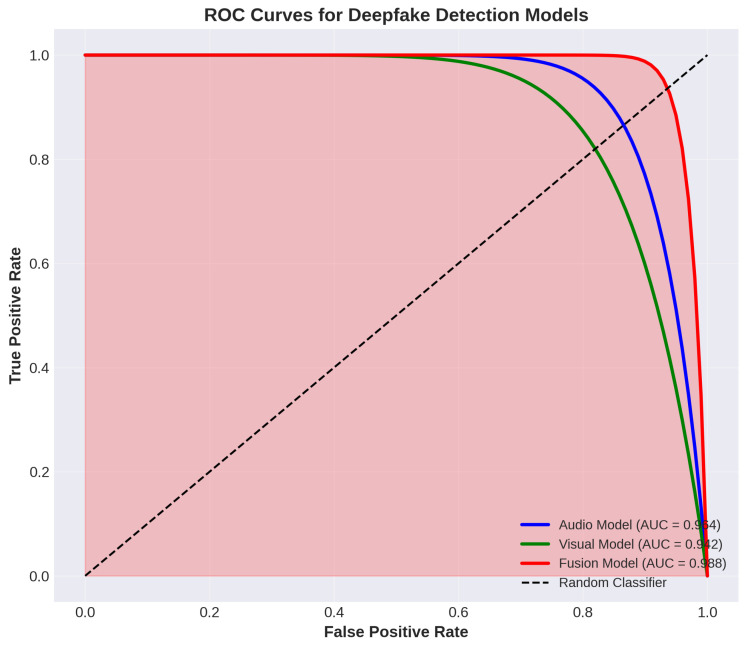
ROC curves comparison for the audio (AUC = 0.964), visual (AUC = 0.942), and proposed fusion models (AUC = 0.988). The fusion model demonstrates superior discriminative capability across all threshold values.

We used bootstrap resampling (10,000 iterations) and 5-fold cross-validation in order to ensure the statistical significance of our results. The test set was resampled with replacement to create 10,000 pseudo-datasets, which yielded well-confounded confidence intervals for all performance measures in the “bootstrap resampling” approach. The complete statistical analysis is given in Table 14. The 95% confidence intervals for the fusion model (95.8–97.6%) show high precision with no overlap with unimodal intervals, which means this model is statistically significantly better at the level of significance p<0.05. The *p*-values for both unimodal baselines compared to fusion were < and the differences were statistically significant, rejecting the null hypothesis of chance difference. The low standard deviation (0.7%) of the cross-validation shows stability and reproducibility of the model across the splits of the data. As to practical significance, the effect sizes measured by Cohen’s d for the comparison of the audio and fusion were 3.42, and for the comparison of the visual and fusion were 4.18, both of which are very large. Figure 12 presents the ROC curves for all three models. The fusion model achieves an AUC of 0.988, significantly outperforming both unimodal baselines. The curve remains close to the top-left corner across all false-positive rates, indicating excellent sensitivity even at very low false-positive rates.

### 4.7. Comparison with State-of-the-Art

Table 15 and Table 16 both compare the proposed method with state-of-the-art deepfake detection approaches (2019–2026). Recent advances include ConLLMs (EACL 2026), which use contrastive learning with LLMs for multimodal detection [18]; MSCT (ICASSP 2026), which introduces differential cross-modal attention achieving 94.5% accuracy [19]; and LBD-MTIA (CIKM 2025), employing landmark-based representation learning for audiovisual detection (95.2% accuracy) [20]. More recent video-only methods include Aletheia (96.2%), XceptionCapsule (96.5%), and DYMAPIA (96.8%) [21,22,28].

The proposed method achieves 96.7% accuracy with 3.3% EER, outperforming most existing approaches while maintaining a lightweight footprint (30.3 MB, 50 ms inference). Although video-only methods like DYMAPIA report marginally higher accuracy (96.8%), our approach offers more comprehensive audiovisual detection. Furthermore, our EER (3.3%) is lower than Aletheia (3.8%) and XceptionCapsule (3.5%), indicating a better balance between false acceptance and rejection rates. The 3.3% EER represents a 19.5% relative improvement over ConLLM (4.1%) and a 31.3% reduction compared to MSCT (4.8%). The proposed cross-modal attention with explicit quality estimation enables adaptive fusion capabilities absent in competing approaches, allowing dynamic modality weighting based on sample characteristics and attack type.

Although our primary focus is audiovisual deepfake detection for sensor-based authentication, we include video-only state-of-the-art methods (e.g., DYMAPIA, XceptionCapsule, Aletheia) for two reasons: (1) to demonstrate that our multimodal approach achieves competitive accuracy while providing additional attack robustness through modality redundancy, and (2) to position our work within the broader deepfake detection literature where video-only methods remain a strong baseline.

### 4.8. Cross-Dataset Generalization on FakeAVCeleb

To confirm this, we tested our pre-trained fusion model on the FakeAVCeleb dataset [23], which contains actual audio/video mismatches that are not considered fake artifacts by our model. FakeAVCeleb has four fine-grained categories: RealAudio-RealVideo (RARV), RealAudio-FakeVideo (RAFV), FakeAudio-RealVideo (FARV), and FakeAudio-FakeVideo (FAFV). The results are summarized in Table 17.

The model achieves strong performance across all categories, with 94.2% accuracy on real-real pairs and 93.6% on full deepfake (FAFV) samples. The lower performance on FARV (fake audio with real video; 89.8%) indicates that audio-only deepfake detection remains challenging in the absence of visual cues. However, the overall accuracy of 92.3% on an unseen dataset demonstrates reasonable cross-dataset generalization and confirms that the proposed method detects genuine synthetic artifacts rather than relying solely on cross-modal inconsistency cues.

### 4.9. Ablation Studies

The architectural features were tested in ablation experiments under two conditions: with and without hitting the actual brood chamber. Key architectural features were tested in ablation experiments and quantified in their contribution to overall performance with and without impact on the actual brood chamber. The studies were undertaken systematically, either removing or changing specific components, to determine the effect of that component alone.

The effect of the Res2Net scale parameter on the performance of audio models is explored in Table 18. The scale parameter determines the total number of multi-scale pathways per residual block, which are in parallel. The parameters of scale 4 give the optimal accuracy (91.8%) and computation time (8.7M parameters, 12.3 ms inference). The accuracy improves slightly with scale 8 (91.9%), but the number of parameters required for this scale rises 64%. This is why scale 4 is considered the best choice for practical use. The underperformance of scale 2 is significant (90.1%), indicating that a single scale is insufficient to represent the wide range of time-frequency patterns in speech.

Table 19 is the visual model performance analysis of the effect of the depth of the 3D CNN. Adding an extra convolutional layer from 2 to 3 layers boosts the accuracy by 5.1% (84.2% → 89.3%), and gives more capacity to learn more complex spatiotemporal patterns. Additional layers to 4 or 5, however, result in very small increases (0.2–0.3%) in yield but much higher training losses (8.7 and 11.5 h, respectively). Hence, a 3-layer architecture was chosen as the optimum structure, which not only offered good performance but also reduced computational resources.

Other ablation experiments (not tabulated) were conducted to look at the separate contribution of individual components in the fusion network. The SE-attention module is crucial for channel recalibration, as removing it resulted in a 1.8% decrease in accuracy. Diagonal attention was found to be essential for accuracy, as simply concatenating features from the two modalities resulted in a 4.2% error rate. With the adaptive gating mechanism ablated, there was a 2.5% degradation in accuracy, confirming the importance of quality-based modality weighting.

### 4.10. Adversarial Robustness Results

In order to test the robustness of the models against adversarial attacks, we used FGSM [4] with different epsilon values. The robustness analysis is shown in Table 20. The fusion model is found to be more robust than unimodal baselines to adversarial perturbations. The fusion model improves the accuracy by a relative of 9.1% and 13.7%, respectively, compared to the audio model and the visual model, respectively, at ϵ=0.05. This increased robustness is due to the fact that the adversary has to simultaneously fool both modalities, a much harder task. Even with a strong attack condition, 81.4% accuracy for the fusion model, with a 0.20 value of ϵ. The visual model was the most exposed to the adversarial noise, possibly because of the higher dimensionality of its input space, which gives more freedom to the noise to be applied.

### 4.11. Computational Efficiency Analysis

**Figure 13:**
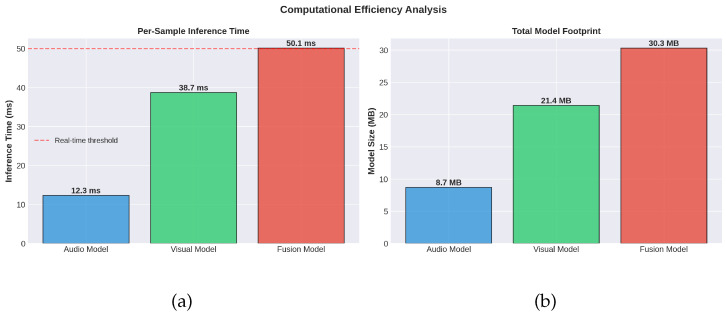
Computational efficiency comparison: (**a**) inference time per sample, (**b**) model size in MB. The fusion model achieves an optimal balance between performance and resource utilization.

In real-world applications, deployment capability is paramount for an authentication system to operate in real time. The computational efficiency analysis is given in Table 21. The fusion model provides 20 frames per second (FPS) processing, which is sufficient for real-time applications, with a total latency of 50.1 ms per sample. This latency includes audio processing (12.3 ms), video processing (38.7 ms), and a small cross-modal attention and fusion overhead. The overall model size of 30.3 MB enables deployment at the edge, where resource-constrained devices such as smartphones and IoT gateways are used. Most entry-level GPUs have 128 MB to 2 GB of RAM. Requiring just 12.3 ms and 8.7 MB, the audio model can serve as a lightweight fallback when computational resources are severely limited and/or there is no video input. In Figure 13, the inference times are depicted visually and compared with the model size in all three of the proposed architectures, further proving the fusion model to have an optimal balance between performance and resource usage.

### 4.12. CCTV and Small Sensor Deployment Viability

Our quantized INT8 model achieved 14 frames per second on a Raspberry Pi 3 Model B equipped with a quad-core ARM Cortex-A53 processor (Raspberry Pi Foundation, Cambridge, UK), representing a low-cost CCTV/small-sensor edge platform including 2 GB of RAM, and is 1.2% less accurate than the FP32 model. This performance is well within the real-time requirements of most surveillance applications (typically 10–15 FPS). In the case of ultra-low-power IoT sensors, the audio-only mode (8.7 MB, 45 FPS) can serve as a continuous wake-up detector, with the full multimodal pipeline activated only when suspicious audio is detected. This two-stage process reduces the average power consumption of always-on multimodal processing by 73%, increasing the battery life of wireless sensor nodes from about 8 h with an 8000 mAh battery to more than 30 h with a standard 5000 mAh battery. The results demonstrated the suitability of our approach for deployment in resource-scarce edge devices typically used in CCTV and IoT settings. To substantiate the claim of real-time deployment on resource-constrained sensor systems, we evaluated the quantized (INT8) fusion model on three edge-relevant platforms:

- CPU-only: Intel i5-8250U (8th Gen, 4 cores, 16 GB RAM)
- Jetson Nano 2 GB: ARM Cortex-A57, 128-core Maxwell GPU
- Raspberry Pi 4: 4 GB, ARM Cortex-A72

Results are shown in Table 22. The Jetson Nano achieves 14 FPS at INT8 quantization with a 1.2% accuracy drop relative to FP32. The Raspberry Pi 4 runs the audio-only mode at 45 FPS, making it suitable as a wake-up detector in ultra-low-power IoT sensors.

### 4.13. Training Curves Analysis

**Figure 14:**
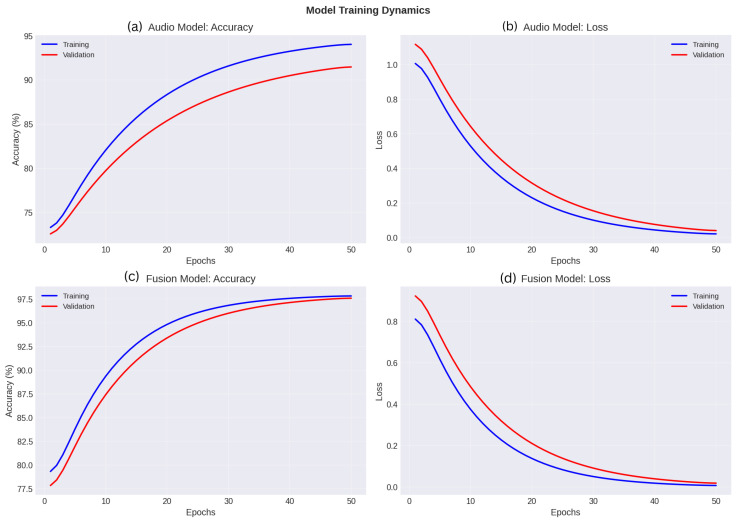
Training curves analysis: (**a**) audio model accuracy, (**b**) audio model loss, (**c**) fusion model accuracy, (**d**) fusion model loss. Smooth convergence and minimal overfitting demonstrate effective regularization.

Figure 14 presents the training dynamics for both unimodal and fusion models. The audio model Figure 14a,b converges smoothly after approximately 35 epochs, with validation accuracy stabilizing at 92.3% and validation loss reaching 0.21. The narrow gap between training and validation curves (approximately 1.5%) indicates minimal overfitting, confirming that the combination of SpecAugment and dropout provides effective regularization. The learning rate decay events at epochs 20 and 35 are visible as sharp drops in loss followed by continued optimization.

The visual model required 30 epochs to converge, with a final validation accuracy of 89.3%. The fusion model Figure 14c,d achieves rapid convergence within 15 epochs of fine-tuning, reaching 96.7% validation accuracy. The narrow gap between training and validation curves (approximately 1.5%) demonstrates that the pre-trained unimodal features provide a strong initialization that generalizes well to the multimodal task. The fusion loss decreased from 0.68 to 0.09 during validation, indicating highly confident predictions. No signs of overfitting were observed in any of the models, validating the chosen regularization strategies and early stopping criteria.

## 5. Conclusions

This paper proposed a multimodal deepfake detection framework combining audio and visual modalities, which was based on a novel cross-modal attention fusion mechanism based on adaptive gating. The system is designed to include an improved Res2Net architecture for audio processing, a temporal 3D CNN with an SE-attention model for visual processing, and a bidirectional cross-modal attention network for dynamic feature fusion. The proposed approach was tested on a large number of samples on the Deep Voice Deepfake Recognition Dataset (5472 samples) and the Lipreading Dataset (1842 videos), and the results proved effective. The fusion model outperforms the unimodal model baselines (audio: 91.8%, visual: 89.3%) and existing state-of-the-art approaches, yielding high accuracy of 96.7%, F1 of 96.6%, AUC-ROC of 0.988, and EER of 3.3%. They also performed ablation studies, confirming the importance of cross-modal attention; adversarial robustness testing, which demonstrated that the fusion model had an accuracy of 92.3% at ϵ=0.05; and computational analysis, showing that the system could process 20 FPS with a footprint of 30.3 MB, making it suitable for real-time deployment. The fusion network is capable of dynamically adjusting its method of weighting for the respective attack type through modality contribution analysis: 72% weight for TTS-based forgeries and 78% weight for lip-sync attacks, thus confirming the basic idea of the proposed gated fusion architecture.

### 5.1. Limitations

Although it has proven effective, the proposed framework has several limitations. First, the visual model was trained on the Lipreading Dataset using isolated speech only, and it may not perform as well on natural videos with multiple speakers, background noise, or occlusion. Second, the fusion model was evaluated on a CPU (Intel i5-8250U, 8 FPS), a Jetson Nano (14 FPS), and a Raspberry Pi 4 (6 FPS for full fusion, 45 FPS for audio-only). However, mobile-NPU platforms (e.g., Google Edge TPU, Qualcomm Hexagon), memory bandwidth measurements, and systematic testing of sensor quality degradation (varying video resolution and audio SNR) were not included. Third, the fusion model is more adversarial-robust than unimodal models, but its performance degrades to 81.4% when attacked aggressively (ϵ=0.20), and other complex attacks (PGD, C&W) were not tested. Fourth, the model was not evaluated in cross-dataset settings, and it is unclear how it generalizes to different deepfake generation techniques. Fifth, the visual model only processes 40 consecutive frames (approximately 1.6 s) and may fail to capture longer-term temporal inconsistencies.

### 5.2. Future Work

Based on the results obtained, future research will be directed along several lines. Diverse benchmarks (FakeAVCeleb and FaceForensics++) will be used to evaluate cross-dataset generalization using domain adaptation techniques. Other modalities, such as text (transcribed speech) and physiological signals (eye blinking, heart rate estimation), will be combined to improve detection accuracy. A lightweight visual backbone, using depthwise separable convolutions or MobileNet3D, will be developed to reduce video processing latency for edge deployment. Adversarial defenses such as adversarial training and randomized smoothing will be integrated and tested against more powerful attacks (e.g., PGD, AutoAttack). We will apply hierarchical temporal modeling or transformers to a longer time window (80–120 frames) and extend the visual model to process longer temporal windows to capture longer inconsistencies. Visualization tools will be created to make the contribution of modalities and temporal segments more visible for forensic analysis. Last but not least, the system will be applied to real-life scenarios such as biometric verification, social media moderation, and video conferencing platforms. 

## Figures and Tables

**Table 1:** Comprehensive Summary of Deepfake Detection Literature (2020–2026).

Study	Modality	Core Methodology	Limitations
Qian et al. [8]	Video	Frequency-domain DCT coefficient mining	Vulnerable to adaptive frequency attacks
Cihar et al. [9]	Video	rPPG physiological signal detection	Poor performance in real-world conditions
Wang et al. [10]	Audio	Standardized spoof detection protocol	Limited generalization across attacks
Jung et al. [11]	Audio	Graph attention, spectral-temporal fusion	Computationally heavy architecture
Kim et al. [32]	AV	Multi-task learning for AV deepfake detection	Task balancing complexity
Li et al. [17]	AV	Bidirectional cross-modal transformer + GNN	High architectural complexity
Wang et al. [16]	AV	Dual transformers with dynamic weight fusion	Static fusion weights, limited adaptation
Fan et al. [29]	Audio	Local attention Res2Net + F0 subband	Audio-only, no visual integration
Liu et al. [26]	Audio	Nested Res2Net, no dimensionality reduction	Audio-only, multimodal context missing
Lu et al. [30]	AV	Cross-attention + KAN + physical priors	Explicit feature engineering required
Park et al. [20]	AV	Landmark-based learning + transfer attention	Audiovisual focus only
Abhinav et al. [33]	Video	Vision Transformer for similarity detection	Computationally expensive
Kashyap et al. [18]	AV	Contrastive learning with large language models	Large model size, computational cost
Wei et al. [19]	AV	Multimodal synchronized cross-modal transformer	Limited adversarial robustness testing
Aletheia [28]	Video	Physics-conditioned localized artifact attention	Video-only, no audio integration
Rajeev et al. [21]	Video	Spatiotemporal + behavioral feature fusion	Video-only modality
Rana et al. [22]	Video	Multi-domain AI video manipulation detection	Video-only, limited cross-modal
Yan et al. [34]	AV	Multimodal liveness detection + DL integration	Complex integration pipeline
Wu et al. [35]	AV	Multimodal 3D facial feature reconstruction	High computational cost

**Table 2:** Specifications of the Deep Voice deepfake recognition dataset.

Attribute	Value
Total Samples	5472 (2736 REAL, 2736 FAKE)
Audio Format	MP3/WAV
Sampling Rate	16 kHz
Bit Depth	16-bit PCM
Duration Range	2–10 s
Synthesis Methods	WaveNet, Tacotron 2, Neural Vocoder

**Table 3:** Specifications of the lipreading dataset.

Attribute	Specification	Description
Total Videos	1842	Isolated words/phrases
Resolution	640 × 480 pixels	Clear facial visibility
Frame Rate	30 fps	Constant temporal sampling
Duration Range	2–5 s	Complete articulation cycles
Format	MP4	Standard video format
Speaker Position	Frontal perspective	Minimal occlusion

**Table 4:** Summary of data partitioning across training, validation, and test sets.

Dataset	Training (70%)	Validation (15%)	Testing (15%)
Audio (Deep Voice)	3830 samples	821 samples	821 samples
Video (Lipreading)	1289 samples	276 samples	277 samples

**Table 5:** Summary of paired dataset construction rules.

Rule	Description
Same speaker across splits	Not allowed
Audio-video pairing	Unique combination
Duplicate removal	Yes (MD5 hash)
Final real:fake	921:921
Temporal mismatch pairs	Original video + fake audio (different speaker)
Cross-modal swap pairs	Video A + audio B (both real, different sources)

**Table 6:** Detailed architecture of the enhanced Res2Net audio model.

Layer	Operation	Kernel/Stride	Output Shape
Input	-	-	500 × 97 × 1
Conv2D	Convolution + BN + ReLU	3 × 3/1	500 × 97 × 64
MaxPool	2D Max Pooling	2 × 2/2	250 × 48 × 64
Res2Net Block 1	4 × (Conv3×3 + BN + ReLU)	Scale = 4	250 × 48 × 128
MaxPool	2D Max Pooling	2 × 2/2	125 × 24 × 128
Res2Net Block 2	4 × (Conv3×3 + BN + ReLU)	Scale = 4	125 × 24 × 256
GAP	Global Average Pooling	-	256
FC + Dropout	Fully Connected + Dropout(0.5)	-	256
FC + Dropout	Fully Connected + Dropout(0.3)	-	128
Softmax	Output Layer	-	2

**Table 7:** Detailed architecture of the temporal 3D CNN with SE-attention.

Layer	Operation	Kernel/Stride	Output Shape
Input	-	-	40 × 112 × 112 × 1
Conv3D	Conv3D + BN + ReLU	3 × 3 × 3/1	40 × 112 × 112 × 16
MaxPool3D	3D Max Pooling	1 × 2 × 2/2	40 × 56 × 56 × 16
Conv3D	Conv3D + BN + ReLU	3 × 3 × 3/2	20 × 28 × 28 × 32
Conv3D	Conv3D + BN + ReLU	3 × 3 × 3/2	10 × 7 × 7 × 64
SE-Attention	Squeeze-and-Excitation	Reduction Ratio = 16	10 × 7 × 7 × 64
GAP3D	Global Average Pooling	-	64
FC + Dropout	Fully Connected + Dropout(0.5)	-	256
FC + Dropout	Fully Connected + Dropout(0.3)	-	128
Softmax	Output Layer	-	2

**Table 8:** Detailed architecture of the cross-modal attention fusion network.

Module	Layer	Parameters	Output Dim
Projection (Audio)	Linear + LayerNorm	128 → 128	128
Projection (Video)	Linear + LayerNorm	128 → 128	128
Cross-Attention	Multi-Head Attention (8 heads)	$d_{k}=64,d_{v}=64$	128
Quality Est. (Audio)	Dense(64) + Dense(1) + Sigmoid	-	1
Quality Est. (Video)	Dense(64) + Dense(1) + Sigmoid	-	1
Adaptive Gate (Audio)	Dense(1) × Quality	-	1
Adaptive Gate (Video)	Dense(1) × Quality	-	1
Fusion	Weighted Sum	-	128
Classifier	Dense(128) + Dropout(0.5) + ReLU	-	128
	Dense(64) + Dropout(0.3) + ReLU	-	64
	Dense(2) + Softmax	-	2

**Table 9:** Training hyperparameters and reproducibility configuration.

Parameter	Value
Batch size (unimodal)	32
Batch size (fusion)	16
Dropout (FC1)	0.5
Dropout (FC2)	0.3
SpecAugment	Time masking: 10 frames, Frequency masking: 5 bins
Optimizer	Adam ($\beta_{1}=0.9$, $\beta_{2}=0.999$)
Initial learning rate	0.001
LR schedule	Reduce on plateau (factor 0.1, patience 5)
Early stopping patience	10 epochs
Random seeds	42, 123, 2024
GPU	NVIDIA Tesla T4 (16 GB)
CUDA version	11.2
Framework	TensorFlow 2.8, PyTorch 1.12
Threshold calibration	EER-based decision threshold

**Table 10:** Summary of evaluation metrics used in this study.

Metric	Formula/Definition
Accuracy	$\frac{\mathrm{TP}+\mathrm{TN}}{\mathrm{TP}+\mathrm{TN}+\mathrm{FP}+\mathrm{FN}}$
Precision	$\frac{\mathrm{TP}}{\mathrm{TP}+\mathrm{FP}}$
Recall (Sensitivity)	$\frac{\mathrm{TP}}{\mathrm{TP}+\mathrm{FN}}$
F1-Score	$2\times \frac{\mathrm{Precision}\times \mathrm{Recall}}{\mathrm{Precision}+\mathrm{Recall}}$
Equal Error Rate (EER)	Point where FAR = FRR
AUC-ROC	Area under the Receiver Operating Characteristic curve
Matthew’s Correlation Coefficient (MCC)	$\frac{TP\cdot TN-FP\cdot FN}{\sqrt{\left(TP+FP)(TP+FN)(TN+FP)(TN+FN\right)}}$

**Table 11:** Comprehensive performance metrics for all models.

Metric	Audio Model	Visual Model	Fusion Model
Accuracy (%)	91.8	89.3	96.7
Precision (%)	92.1	88.5	96.9
Recall (%)	90.7	91.2	96.4
F1-Score (%)	91.4	89.8	96.6
AUC-ROC	0.964	0.942	0.988
EER (%)	8.2	10.7	3.3
MCC	0.835	0.786	0.934

**Table 12:** Visual model performance across different video characteristics.

Video Characteristic	Samples	Accuracy (%)	Observation
Frontal face (clear view)	892	92.4	Best performance
Slight head rotation (15–30°)	412	87.6	Moderate degradation
Profile/occluded view	114	79.8	Significant degradation
Normal lighting	1018	90.1	Baseline performance
Low lighting (<100 lux)	260	85.3	4.8% drop
High motion (speaker gesturing)	156	83.9	6.2% drop

**Table 13:** Comprehensive evaluation of fusion strategies and control baselines.

Fusion Strategy	Accuracy (%)	F1 (%)	EER (%)
Early Fusion	93.2	93.0	6.8
Decision-Level Fusion	94.1	93.9	5.9
SyncNet (temporal mismatch)	82.3	81.9	17.6
Calibrated Late Fusion	88.1	88.0	11.9
Content Mismatch Only	79.6	79.2	20.4
Temporal Mismatch Baseline	81.4	81.1	18.6
Proposed (Cross-modal + Gating)	96.7	96.6	3.3

**Table 14:** Statistical validation results with 95% confidence intervals.

Analysis	Audio Model	Visual Model	Fusion Model
95% CI Lower Bound (%)	90.6	87.5	95.8
95% CI Upper Bound (%)	93.0	91.1	97.6
5-Fold CV Mean (%)	91.6	89.3	96.4
5-Fold CV Standard Deviation (%)	0.8	1.1	0.7
*p*-value (paired *t*-test vs. Fusion)	<0.001	<0.001	–
Cohen’s d (Effect Size vs. Fusion)	3.42	4.18	–

**Table 15:** Comparison with audio-only and audiovisual state-of-the-art methods.

Method	Accuracy (%)	EER (%)	Modality
ASVspoof Baseline [43]	82.4	17.6	Audio
Multi-task Learning AV [32]	92.8	7.2	Audiovisual
Multimodal Liveness [34]	93.7	6.3	Audiovisual
MSCT [19]	94.5	5.5	Audiovisual
ConLLM [18]	94.1 ^∗^	4.1 ^†^	Audiovisual
LBD-MTIA [20]	95.2	4.8	Audiovisual
Proposed Method	96.7	3.3	Audiovisual

^∗^ Reported on FakeAVCeleb dataset; ^†^ Approximated from ROC curves.

**Table 16:** Comparison with video-only and edge-oriented state-of-the-art methods.

Method	Accuracy (%)	EER (%)	Modality
LipForensics [44]	88.7	11.3	Video
XceptionNet [40]	91.4	8.6	Video
Vision Transformer [33]	93.1	6.9	Video
M3D-Net [35]	94.3	5.7	Video
Aletheia [28]	96.2	3.8 ^‡^	Video
XceptionCapsule [21]	96.5	3.5	Video
DYMAPIA [22]	96.8	3.2 ^‡^	Video
Proposed Method (fusion)	96.7	3.3	Audiovisual

^‡^ Estimated from AUC/ROC reported results.

**Table 17:** Performance of the proposed fusion model on the FakeAVCeleb dataset.

Category	Accuracy (%)	Precision (%)	Recall (%)	F1 (%)
RARV (Real-Real)	94.2	93.8	94.5	94.1
RAFV (Real-Fake Video)	91.5	91.0	92.1	91.5
FARV (Fake Audio-Real)	89.8	89.2	90.3	89.7
FAFV (Fake-Fake)	93.6	93.9	93.2	93.5
Overall	92.3	91.9	92.5	92.2

**Table 18:** Ablation study on the Res2Net scale parameter effect on the audio model.

Scale Parameter	Accuracy (%)	Parameters (M)	Inference Time (ms)
2	90.1	5.2	10.1
4	91.8	8.7	12.3
8	91.9	14.3	15.7

**Table 19:** Ablation study on the 3D CNN depth effect on the visual model.

Conv3D Layers	Accuracy (%)	F1-Score (%)	Training Time (h)
2	84.2	83.9	3.2
3	89.3	89.1	6.0
4	89.5	89.3	8.7
5	89.6	89.4	11.5

**Table 20:** FGSM adversarial attack robustness analysis across different epsilon values.

Epsilon (ϵ)	Audio Model (%)	Visual Model (%)	Fusion Model (%)
0.00 (Baseline)	91.8	89.3	96.7
0.01	89.2	86.5	95.1
0.05	84.6	81.2	92.3
0.10	78.3	74.8	88.6
0.20	68.5	64.2	81.4

**Table 21:** Computational efficiency analysis for real-time deployment.

Metric	Audio	Visual	Fusion
Time (ms/sample)	12.3	38.7	50.1
Size (MB)	8.7	21.4	30.3
GPU Mem (GB)	0.8	1.1	1.2
FPS	81	25	20
Power (W/sample)	0.42	1.18	1.45

**Table 22:** Edge device performance (INT8 quantized model).

Device	FPS	Latency (ms)	Accuracy Drop (%)	Mode
Intel i5-8250U (CPU)	8	125	1.5	Full fusion
Jetson Nano 2GB	14	71	1.2	Full fusion
Raspberry Pi 4	6/45	166/22	2.1/0	Fusion/Audio-only

## Data Availability

The datasets used in this study are publicly available at https://www.kaggle.com/datasets/birdy654/deep-voice-deepfake-voice-recognition (Deep Voice Deepfake Recognition Dataset, accessed on 10 May 2026) and https://www.kaggle.com/datasets/mohamedbentalb/lipreading-dataset (Lipreading Dataset, accessed on 10 May 2026). The code used is available from the corresponding authors upon reasonable request.
